# Energy audit and associated carbon footprint estimation for a Meta Abo brewery

**DOI:** 10.1016/j.heliyon.2024.e28300

**Published:** 2024-03-16

**Authors:** Eba Adino, Mikiyas Abewaa, Amare Tiruneh

**Affiliations:** a Department of Environmental Engineering, Addis Ababa Science and Technology University, 16417, Addis Ababa, Ethiopia; b Department of Chemical Engineering, School of Mechanical, Chemical and Materials Engineering, Adama Science and Technology University, Adama, Ethiopia; c Department of Chemical Engineering, College of Engineering and Technology, Wachemo University, Hossana, Ethiopia

**Keywords:** Brewery industry, Carbon footprint, Energy audit, Energy loss, Energy saving, Energy efficiency

## Abstract

Industrial development in Ethiopia is rapidly increasing, leading to a growing gap between energy supply and demand. To address this, efficient energy utilization in existing industries is crucial. Energy audits identify energy losses and recommend saving measures. Therefore, this study evaluates energy efficiency through an audit and estimates greenhouse gas emissions for a Meta Abo brewery. The indirect method of energy audit and the greenhouse gas protocol of carbon footprint estimation were used to evaluate the energy efficiency and carbon emissions of Meta Abo brewery. The boiler efficiency for Bono Energia and Cochran boilers was found to be 79.2% and 80.9%, respectively. Poor insulation caused an estimated annual fuel loss of 35,350 l (638,768 Ethiopian birr) for steam pipes, while steam leakage resulted in a loss of 31,614 l (571,265 Ethiopian birr). The factory's high electricity expense was attributed to a diesel generator consuming 6000 l/d. Greenhouse gas emissions raised from 9156 to 22,697 tons of CO_2_ equivalent between 2014 and 2018. Implementing the proposed energy-saving measures could save 20.4 TJ of thermal and electrical energy annually, costing approximately 8.5 million Ethiopian birr, and reduce boiler emissions by 455 tons of CO_2_ equivalent. Therefore, implementation of these measures is recommended.

## 1 Introduction

Energy is the lifeblood of modern economies, driving economic growth and supporting industrial development [1]. It encompasses different forms like electricity, fossil fuels, and other renewable sources [2]. Energy plays a pivotal role in powering industries, transportation, and residential sectors, enabling human activities and economic productivity [3]. The global demand for energy is escalating, with projections indicating a steady increase in consumption in the years ahead. The International Energy Agency (IEA) forecasts a 1.3% annual increase in global energy demand from 2020 to 2040 [4]. This surge is driven by factors such as population growth, urbanization, and economic progress in emerging economies. Consequently, countries worldwide face the challenge of meeting future energy requirements while minimizing costs and environmental impacts [5]. To address future energy needs while mitigating costs and environmental consequences, it is crucial to have a comprehensive understanding of energy supply and consumption patterns [6,7]. However, the rising energy consumption has associated environmental implications. The burning of fossil fuels remains the primary contributor to global energy production, resulting in approximately three-quarters of greenhouse gas emissions [8]. These emissions contribute to climate change, leading to increasing temperatures, rising sea levels, extreme weather events, and other adverse environmental effects [9,10]. To mitigate these impacts, countries around the world are embracing renewable energy sources such as solar, wind, hydro, and geothermal power. Renewable energy technologies offer the advantages of being clean, abundant, and sustainable. They aid in reducing greenhouse gas emissions, improving air quality, and enhancing energy security through diversification of the energy mix [11]. Achieving a sustainable energy future necessitates a transition to low-carbon and renewable energy systems, coupled with energy efficiency measures. This transition presents both challenges and opportunities for countries to enhance their environmental performance, reduce reliance on fossil fuels, and foster economic growth through the development of clean energy technologies [[12], [13], [14]].

With its rapid economic expansion and burgeoning population, Ethiopia has witnessed a notable surge in energy requirements. Energy consumption in Ethiopia spans across diverse sectors, encompassing residential, commercial, transportation, and industrial domains. The industrial sector emerges as a prominent energy consumer, constituting a substantial proportion of the nation's overall energy usage [15]. As per the Ethiopian Energy Authority (EEA), the industrial sector accounted for approximately 30% of the total electricity consumption in the country in 2020. This heightened energy demand within the sector stems from various industries such as manufacturing, mining, and construction [16]. Among these rapidly expanding industries, the beverage sector, including breweries, occupies a significant position due to its energy-intensive operations [17]. To foster industrial growth and development, it becomes imperative to ensure a reliable and cost-effective energy supply while concurrently promoting energy efficiency measures. Ethiopia has been actively engaged in diversifying its energy sources and augmenting its electricity generation capacity [18]. The government's commitment to achieving universal electricity access by 2025 and increasing the share of renewable energy in the energy mix demonstrates Ethiopia's determination to meet its energy needs sustainably [19].

Ethiopia's industrial sector has been experiencing rapid expansion, making it one of the key drivers of the country's economic growth. The government's focus on industrialization and attracting foreign direct investment has led to the establishment and growth of various industries. These industries, including the beverage sector, have contributed to job creation, export promotion, and overall economic development [20]. However, the expansion of industries and the corresponding increase in energy consumption raise concerns about greenhouse gas emissions and environmental impact. It is crucial to focus on energy conservation measures to reduce energy costs and minimize the industry's carbon footprint. Meta Abo Brewery, as a prominent player in Ethiopia's brewing industry, plays a significant role in meeting the country's growing demand for beverages [21]. The brewery's substantial production capacity requires a significant amount of energy, making energy consumption a critical aspect of its operations. The brewing process, which involves heating, cooling, and fermentation, requires significant energy inputs [22]. As the beverage sector continues to grow, addressing energy consumption and implementing energy conservation measures becomes crucial to ensure sustainable industrial development [23]. By doing so, Ethiopia can balance its industrial growth with environmental sustainability and work towards achieving its development goals.

Energy conservation plays a vital role in sustainable industrial development. It refers to the deliberate and systematic efforts to reduce energy consumption without compromising productivity and quality [24]. Assessing energy efficiency and conducting energy audits are essential steps in identifying opportunities for improvement [25]. It involves evaluating energy use patterns, identifying energy-saving technologies and practices, and implementing measures to optimize energy consumption [26]. Conducting energy audits and assessments can help identify energy-intensive processes, energy losses, and areas for improvement [27]. By optimizing production processes, reducing energy waste, and implementing process modifications, manufacturing industries can achieve significant energy savings [28]. Previous research studies have highlighted the significance of energy conservation measures in industries. These studies have explored technology upgrades, process optimization, and the implementation of energy management systems as effective ways to enhance energy efficiency, reduce operational costs, and mitigate environmental impacts. Additionally, the financial and policy aspects related to energy conservation have been subjects of research, aiming to identify financing mechanisms, incentives, and supportive policy frameworks [29].

Inefficient energy practices within industries contribute to the release of greenhouse gas emissions, leading to an amplified carbon footprint. The carbon footprint represents the overall quantity of greenhouse gases, predominantly carbon dioxide (CO_2_), emitted directly or indirectly by an individual, organization, or product. Comprehending the correlation between energy consumption and the carbon footprint plays a pivotal role in effectively addressing environmental concerns [[30], [31], [32]]. Previous studies have examined energy consumption in industries; however, they often fail to establish a connection with the carbon footprint, resulting in a research gap regarding the understanding of the environmental impact of energy usage. Grasping the relationship between energy consumption and the carbon footprint is essential for formulating efficient strategies to reduce greenhouse gas emissions [33]. Nevertheless, there exists a research gap when it comes to specifically examining this relationship within Ethiopian manufacturing industries. Previous research has predominantly focused on assessing energy usage from a cost-saving perspective while overlooking environmental considerations. Bridging this research gap holds paramount importance in gaining insights into the carbon emissions stemming from energy consumption within Ethiopian manufacturing industries. This study aims to fill these gaps by conducting comprehensive energy audits to identify opportunities for energy conservation and estimate the potential reduction in the carbon footprint through the implementation of energy-saving measures.

## 2 Materials and methods

### 2.1 Description of study area

**Fig. 1:**
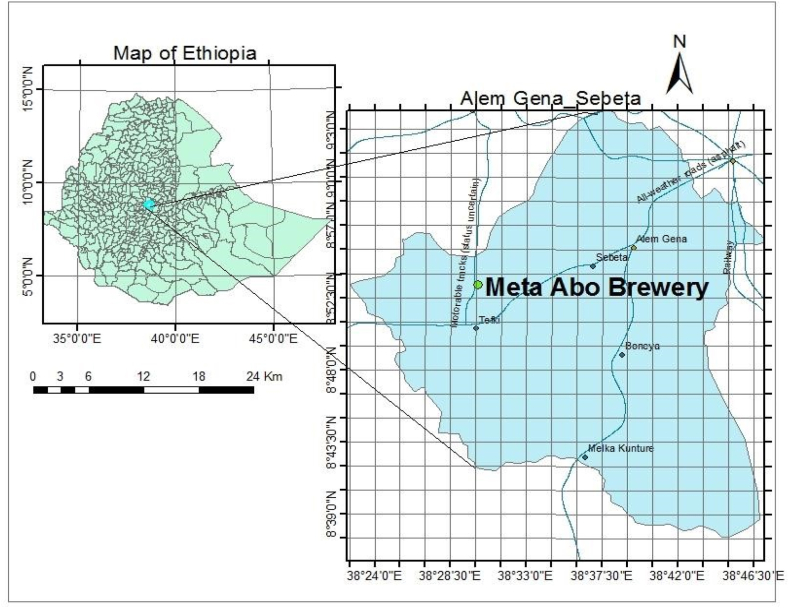
Study area.

Meta Abo Brewery is situated in the town of Sebeta. The town is positioned 27 km southwest of the capital Addis Ababa in the Oromia Region of Central Ethiopia. Geographically, the company is located at an average elevation of 2182 m above sea level to the south of Mount Mogle. The exact coordinates are 8° 54.68′ North latitude and 38° 55.72′ East longitude, as depicted in Fig. 1. DIAGEO acquired and took ownership of the brewery in 2013, undertaking a comprehensive rehabilitation that resulted in an increased annual capacity of 1.6 million hectolitres and the introduction of four new products. The current beer brands produced by the brewery are Meta beer, Malta Guinness, Kurumalt, Guinness, and Guinness beer. The factory operates 24 h a day. Recently, Meta Abo was sold to Castel Group, a multinational beverage company that has solidified its dominance in the Ethiopian market by acquiring a competitor's brewery. The brewery relies exclusively on fuel as its energy source. The research was conducted from October 1st, 2018, to March 31st, 2019, at the premises of Meta Abo Brewery S.C. A combination of exploratory and case study methodologies was employed.

### 2.2 Instruments used

The pH level of the boiler water was assessed using a pH meter (HI99141). To measure temperatures remotely, an infrared thermometer (Digital IR Infrared, China) utilizing infrared technology was employed. On-site, a conductivity meter (CCT-3320 V, China) was utilized to gauge the electrical conductivity of the water sample. An ultrasonic flow meter (FDT-40, UK) equipped with ultrasonic sensors mounted on pipelines was employed to measure the flow of liquids through pipelines of varying sizes. During the energy audit of motors and compressors, voltages and currents were measured using a Digital Multi-meter (Fluke 175, USA). To identify air leakages in the compressed air distribution lines, a leak detector (Ultra probe 100S, India) was utilized. Flue gas analysis was conducted by sampling and analyzing the flue gas using a portable combustion analyzer (E4400, Germany).

### 2.3 Baseline energy performance

To gather historical data on energy consumption, an initial examination of the location was carried out, which involved site visits, walkabouts, and an on-site investigation of all unit operations and processes. Data from the past five years (2014–2018) regarding production and energy performance were collected using official production records, maintenance and operation procedures, and energy bills.

### 2.4 Energy audits

The boiler fuel utilized is furnace oil obtained from Total Ethiopia, which possesses the following specifications: carbon (83%), hydrogen (12.15%), oxygen (0.9%), nitrogen (0.4%), sulphur (3.0%), and moisture content (0.55%). This fuel exhibits a gross calorific value of 43,167 kJ/kg. To determine the efficiency of the two boilers, indirect methods of boiler testing were employed, as illustrated in Equations (1), (2), (3), (4), (5), (6), (7), (8), (9), (10), (11), (12), (13), (14), (15), (16)). By utilizing indirect approaches, the percentage of energy losses is deducted from 100% to determine the overall energy efficiency [34,35]**.** The boilers specification is presented in Table 1.

**Table 1:** Specification of Boiler number 1 (BONO ENERGIA) and Boiler number 2(COCHRAN).

S/N	Specification	Boiler 1	Boiler 2
1.	Manufacturer/Country	BONO ENERGIA/Italy	COCHRAN/Scotland
2.	Type	Fire tube	Fire tube
3.	Year of manufacture	2008	2010
4.	Design pressure	15 bar	15
5.	Maximum working pressure	7.3 bar	7.5
6.	Maximum allowable Temperature	202 °C	–
7.	Steam Capacity (Rated output)	10 tons/h	12000 kg/h.
8.	Serial Number	9249	35/5920
9.	Volume	15,000 lit	–
10.	Length of boiler	5.8 m	6 m
11.	Diameter of boiler	2.95 m	3 m
12.	Average boiler room temp.	30 °C	30 °C
13.	Average surface temperature of boiler head	63.6 °C	61 °C
14.	Average surface temperature of boiler side	39.5 °C	38 °C
15.	Flue gas temperature	229 °C	200 °C
16.	Percentage of CO_2_ in flue gas	12.5 %	12.7 %
17.	Percentage of O_2_ in flue gas	4.7 %	4.6 %

Loss of heat due to dry flue gas (L_1_)

(1) $$L_{1}\left(\%\right)=\frac{m*c_{p}x\left(T_{f}-T_{a}\right)}{\text{GCV}}$$

(2) $$m\left(kg\right)=\left(\frac{mass\;of\;actual\;air\;supplied}{kg\;of\;fuel}\right)+mass\;of\;fuel\;supplied$$

(3) $$L_{2}\left(\%\right)=\frac{9*H_{2}*\left(584+C_{p}\left(T_{f}-T_{a}\right)\right)}{GCVoffuel}*100$$

(4) $$L_{3}\left(\%\right)=\frac{M*\left(584+C_{p}\left(T_{f}‐T_{a}\right)\right)}{GCVoffuel}*100$$

(5) $$L_{4}\left(\%\right)=\frac{\text{AAS}*\text{Humidityfactor}*C_{p}*\left(T_{f}-T_{a}\right)}{\text{GCV}}*100$$

(6) $$Q_{rad}\left(\text{watt}\right)=\sigma \;*\;e*\;A\;*\;\left(T_{s1}^{4}\;–\;T_{a}^{4}\;\right)$$

(7) $$L_{5}\left(\%\right)=\frac{Q_{rad}}{GCV*fuel\;flow\;rate}*100$$

(8) $$Q_{\text{conv}}\left(\text{watt}\right)=h_{conv}*A_{s}*\;\left(T_{s}-T_{a}\right)$$

(9) $$L_{6}\left(\%\right)=\frac{Qconv}{GCV*fuel\;flow\;rate}*100\%$$

(10) $$L_{7}\left(\%\right)=\frac{{\overset{\cdot }{m}}_{blowdown}\left(h_{blowdown}-h_{makeup}\right)}{{\overset{\cdot }{m}}_{fuel}\;GC}*100$$

where L_1_ is the loss of heat due to dry flue gas, L_2_ is the loss of heat due to the presence of H_2_ in the fuel, L_3_ is the heat loss due to evaporation of moisture present in fuel, L_4_ is the heat loss due to moisture present in the air, L_5_ is Loss of heat by surface radiation, L_6_ is the loss of heat by surface convection, L_7_ is the heat loss due to boiler blow down, m is the total mass of flue gas (kg), GCV is a gross calorific value (kJ/kg), H_2_ is the percentage of H_2_ in fuel, Cp is the Specific heat of steam (1.89 kJ/kg ^0^C), T_f_ is Flue gas temperature (°C ^o^C), T_a_ is Ambient temperature (°C), 584 is the latent heat corresponding to the partial pressure of water vapor and is equal to 2452.8 kJ/kg, M is mass of moisture in 1 kg fuel, AAS is the actual mass of air supplied per Kg of fuel (17.76 kg), Q conv is convection heat loss, Q rad is the radiation heat loss, σ is Stefan-Boltzmann constant (5.67 × 10^−8^ W/m^2^ K^4^), ε is the emissivity (0.9), A is the area of the boiler surface (m^2^), and T_S1_ is the surface temperature of the boiler head (K).

Steam table, thermodynamic table, and psychometric chart were used to estimate process values at measured temperatures and pressures.

(11) $$Boiler\;Efficiency\;\left(\eta \right)=100-\left(L_{1}+L_{2}+L_{3}+L_{4}+L_{5}+L_{6}+L_{7}\right)$$

The pressure drop per unit length of the steam line is given by

(12) $$\Delta P\;\left(\text{bar}\right)=\frac{P1-P2}{L}$$

where ΔP is pressure loss in the bar, P_1_ is the pressure at the boiler house in the bar, P_2_ is the pressure at end use in the bar and L is pipeline length in meters.

For surface temperature up to 200 °C the heat loss can be calculated using the following equations [36,37].

(13) $$QT\left(kcal/hr.m^{2}\right)=A\left(\frac{\left[10+\left(Ts-Ta\right)\right]}{20}\right)\left(Ts-Ta\right)$$

where Q_T_ is total heat loss in kcal/hr.m^2^, A is surface area of pipe in m^2^, T_s_ is pipe surface temperature in ^0^C and Ta is Ambient temperature (24 °C).

(14) $$\text{Estimated}\;\text{equivalent}\;\text{fuel}\;\text{loss}\;\left(\text{kg}\right)=\frac{Q_{T}\;x\;\text{Yearly}\;\text{hours}\;\text{of}\;\text{operation}}{\text{GCV}\;x\;\text{Efficiency}\;\text{of}\;\text{Boiler}}$$

The gross estimate of steam loss through steam leakage is calculated using Napier's equation [38,39].

(15) $$Q_{leak}\left(\text{kJ}/\text{hr}\right)={\overset{\cdot }{m}}_{leak}\left(h_{leak}-h_{makeup}\right)$$

where Q_leak_ is steam leak heat loss in kJ/hr, m_leak_ is steam leak mass flow rate in kg/hr, h_leak_ is the enthalpy of the steam leak in KJ/kg and h_makeup_ is the enthalpy of makeup water in KJ/kg.

(16) $$\text{Estimated}\;\text{total}\;\text{fuel}\;\text{loss}\;\left(\text{kg}\right)=\left(\frac{Q_{\text{leak}}\;x\;\text{Yearly}\;\text{hours}\;\text{of}\;\text{operation}}{\text{GCV}\;\left(\frac{\text{kJ}}{\text{kg}}\right)\;x\;\text{Efficiency}\;\text{of}\;\text{Boiler}}\right)$$

### 2.5 Carbon footprint estimation

To calculate the carbon footprint in terms of CO_2_ equivalent (Eq), established methodologies and recommendations outlined in IPCC 2006 [40,41] were adhered to. The greenhouse gas (GHG) emissions, measured in tons of CO_2_ eq, were determined using the formula:

(17) $${\text{GHG}\;\text{Emission}\;\text{as}\;\text{ton}\;\text{of}\;\text{CO}}_{2\text{eq}}=\left(\text{Amount}\;\text{of}\;\text{fuel}\;\text{used}\right)\;*\;\left(\text{NCV}\right)*\left(\text{Emission}\;\text{factor}\;\right)$$

Here, NCV signifies the gross caloric value of the fuel in GJ/ton. As specific emission factors for the country were unavailable, default emission factors specified in the Tier carbon footprint estimation were employed.

## 3 Results and discussions

### 3.1 Energy consumption data

The energy consumption data from 2014 to 2018 for Meta Abo Brewery is provided in Table 2.

**Table 2:** Energy data for Meta Abo brewery from years 2014–2018.

Year	Amount of Electricity (kWh) from national grid	Amount of fuel oil Generator (L)	Amount of fuel for Boilers (L)	Production Volume (HL)
**2014**	7,262,230.00	–	3,075,266.00	716,672.00
**2015**	8,701,477.00	–	3,544,140.00	814,745.00
**2016**	–	3,026,859.00	3,126,809.00	878,450.00
**2017**	–	3,789,230.00	3,656,453.00	925,647.00
**2018**	–	4,001,235.00	3,837,092.00	932,602.00

Until 2015, the factory sourced its electricity supply from the Ethiopian Electric Power Corporation (EEPCO). However, due to power interruptions from EEPCO, the factory currently relies primarily on electricity generated from a diesel generator. The daily consumption of diesel fuel by the generator exceeds 6000 L, while the two boilers consume approximately 9000 L of light fuel oil. This substantial fuel consumption has resulted in a significant increase in the factory's expenses. Total Ethiopia Oil Company serves as the supplier of fuel oil for the factory.

As indicated in Table 3, there was a notable rise in the energy expenses for the brewery following the complete transition to onsite generation after 2015. The Specific Energy Consumption (SEC), which represents the energy consumption relative to production during the corresponding period, demonstrated an upward trend. Table 4 further illustrates the increasing patterns observed in both specific energy consumption and specific fuel consumption.

**Table 3:** Energy cost data of Meta Abo Brewery (2014–2018).

Year	Cost of electricity and fuel for generator (Birr)	Cost of fuel oil for the boiler (Birr)
**2014**	1,053,023.00	55,570,048.00
**2015**	26,400,000.00	54,223,588.00
**2016**	37,988,600.00	54,695,342.00
**2017**	55,023,798.00	67,827,217.00
**2018**	53,325,450.00	72,022,230.00

**Table 4:** Specific energy consumption(SEC) of Meta Abo Brewery.

Year	Specific electricity from the grid, (kWH)/hL of beer	Specific fuel, (L)/hL of beer	Specific energy, (MJ/hL)
2014	10.13	4.29	201.89
2015	10.68	4.35	206.13
2016	–	6.89	265.65
2017	–	8.04	310.07
2018	–	8.68	334.76

The initial step in assessing energy performance involves the analysis of historical energy consumption and costs. This process involves organizing billing details and establishing a foundation for conducting a more comprehensive examination of energy performance. It allows for internal comparisons between different time periods, locations, and production units, as well as external comparisons against industry-specific performance standards. For Meta Abo Brewery, the average specific fuel consumption was recorded as 6.45 L per hectoliter (L/hL), while the average electricity consumption stood at 10.45 kW-hours per hectoliter (kWh/hL).

**Fig. 2:**
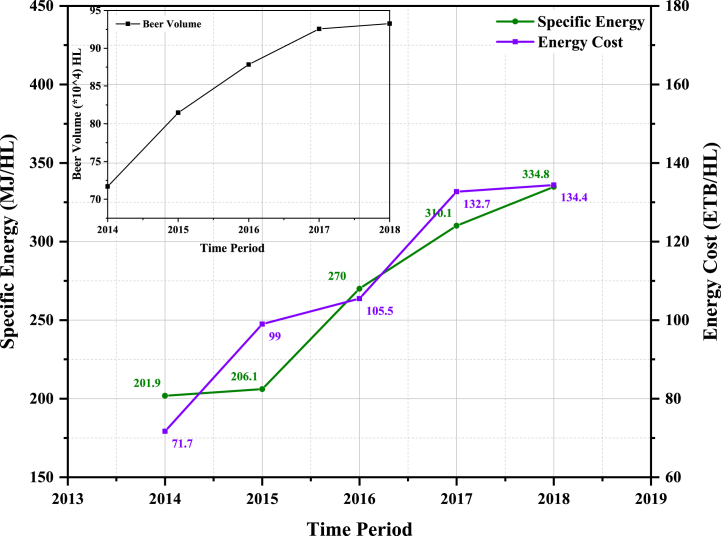
Trend in energy utilization and cost at Meta Abo Brewery.

Fig. 2 illustrates the presence of a range of variations, allowing for external benchmarking. Best brewing practices serve as reference points for specific energy usage achievable through optimal operational practices and brewery equipment. According to Carbon Trust, world-class brewers have achieved a specific fuel oil consumption of 3.9 L per hectoliter (L/hL) and a specific electricity consumption of 7.7 kW-hours per hectoliter (kWh/hL) [42,43]. On average, there is a potential for approximately 39% reduction in fuel oil consumption. The specific energy trend has shown a significant increase since 2015, attributed to the complete shift from electricity supplied by the Ethiopian Electric Power Corporation to onsite generation, as depicted in Fig. 2. This shift is driven by inefficiencies in energy generation and distribution systems. Consequently, the energy cost has been on the rise due to increasing fuel purchase costs and higher production volumes throughout the year.

### 3.2 Energy audit results

#### 3.2.1 Boilers performance evaluations

**Fig. 3:**
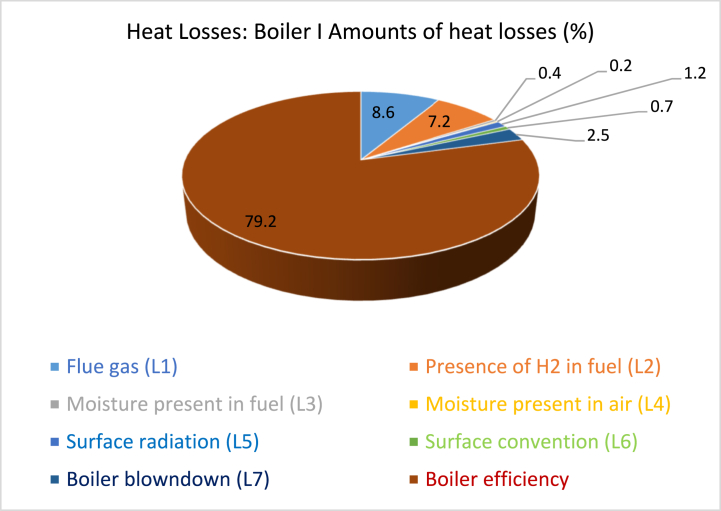
Summary of heat losses in Boiler I for Meta Bo brewery (Bono Energeia).

**Fig. 4:**
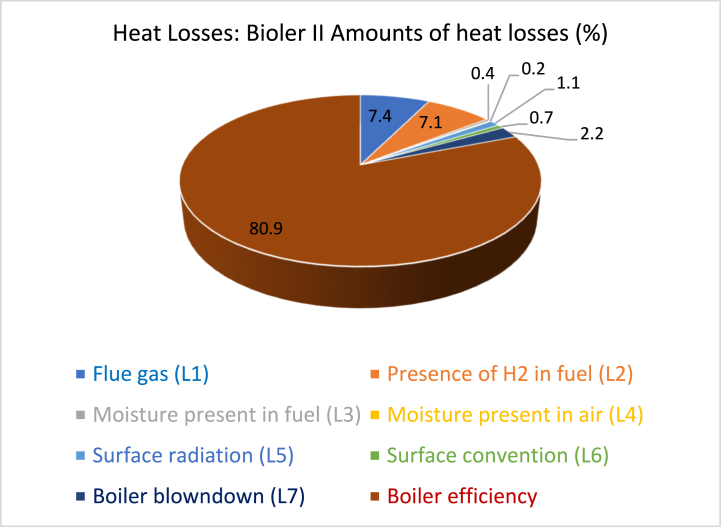
Summary of heat losses in Boiler II for Meta Abo brewery (Coachran).

The heat losses in the first and second boilers are depicted in Fig. 3, Fig. 4 respectively.

Among the various losses encountered, the energy losses attributed to dry flue gas, hydrogen presence in the fuel, and blowdown stand out as significant, prompting the need for proactive measures to mitigate them. In comparison to the standard efficiency observed in similar fire tube boilers, Boiler I operates at a lower efficiency level, which typically ranges around 85–87% [38,44].

Based on the computations, it is evident that there are notable energy losses resulting from dry flue gas, the presence of hydrogen in the fuel, and blowdown. These losses have prompted the implementation of measures to actively reduce them. Additionally, Boiler II is operating at a lower efficiency level when compared to the standard efficiency typically achieved by similar fire tube boilers, which can reach approximately 85–87% [45,46].

#### 3.2.2 Energy audits in steam distribution lines

The majority of steam pipelines at Meta Abo Brewery have been equipped with insulation. Specifically, the steam pipelines within the boiler room have been adequately insulated. There is a new, well-insulated line connecting the boiler room to the new bottling section. However, other lines utilize existing steam pipes that suffer from insulation defects. Poorly insulated pipe sections are particularly prevalent in various areas, notably the brew house. The boiler's average outgoing steam pressure measures 6.8 bar, while the pressure at the brew house stands at 4.89 bar. The total heat loss resulting from inadequate insulation and the estimated fuel loss equivalent for other steam lines have been calculated and are presented in Table 5.

**Table 5:** Estimated equivalent fuel loss due to poor insulation in Meta Abo Brewery.

Line Description	Temp. With allowable pressure drop(°C)	Total heat loss (KJ/hr)	Estimated equivalent fuel loss (L)
Boiler Room – Brew House	163.8	39,969.83	11,225.00
Boiler Room – New bottling	159.8	15,909.15	4467.77
Boiler Room – Old bottling	163.	53,221.54	14,945.20
Boiler Filtration and Draught	156.2	16,780.56	4711.70
**T Total**			**35,349.67**

Instances of steam leakage were identified at the Brew house, Filtration room, and Bottling section, primarily caused by the neglect of maintenance practices. These leakages were a result of pipe, pipe fitting, and valve failures. To quantify the impact, the total fuel loss for each observed steam leakage was calculated and presented in Table 6.

**Table 6:** Estimated equivalent fuel loss due to steam leakage in Meta Abo Brewery.

Leakage location	Approximate Hole Diameter (inches)	Steam pressure in (Psi)	Mass flow of the steam leak (lb/hr)	Enthalpy of steam in (KJ/kg)	Total estimated heat loss (KJ/hr)	Estimated total fuel loss (kg/year)
Brew House	0.08	85.14	22.01	2746	58,219.86	11,801.23
Filtration Room	0.08	58.58	15.14	2738	39,926.53	8093.15
Old Bottling section	0.08	60.32	15.59	2739	41,128.8	8336.86
Total						**28,231.24**

#### 3.2.3 Identified energy saving measures

Managing the energy efficiency and atmospheric emissions of a boiler system primarily relies on controlling excess air. The achievement of perfect combustion necessitates a precise amount of O2, while additional (excess) air is required to ensure complete combustion. However, an excessive amount of excess air leads to heat and efficiency losses [37]. By reducing the excess air in Boiler I from 27.3% to the appropriate level of 15%, a loss reduction of approximately 1.8% can be achieved. Similarly, decreasing the excess air in Boiler II from 28.1% to the optimal excess air percentage of 15% will result in a loss reduction of around 1.9%.

Utilizing the heat from flue gases presents an opportunity to preheat the boiler feed water using an economizer [44,47]. Information obtained from boiler manufacturers' literature reveals that a standard non-condensing boiler typically has a flue gas temperature of 135 °C. Considering the stack temperature of 229 °C and the feed water inlet temperature of 97 °C, it is feasible to install an economizer that aligns with these temperature requirements. Therefore, it is advisable to harness the waste heat emitted from the boiler chimney and employ an economizer to preheat the boiler feed water.

A majority of the steam pipes at Meta Abo Brewery have insulation, but there are specific pipe sections that require re-insulation to minimize energy waste. The total length of these pipes that need re-insulation is approximately 230 m. Additionally, ensuring proper maintenance of the steam distribution systems is crucial to prevent steam leakage and reduce energy waste.

During the brewing process, wort boiling consumes a significant amount of energy. However, there is currently no heat recovery system in place for the wort kettles at Meta Abo Brewery. The use of live steam for wort boiling presents an opportunity for energy recovery through the implementation of a wort pre-heater. By incorporating a plate-type heat exchanger as a wort pre-heater, substantial energy cost savings can be achieved, as the recovered heat can be effectively utilized. It is proposed to equip the two wort kettles with wort pre-heaters to enhance efficiency.

The steam temperature reaching the brew house is approximately 15 °C, while the temperature of the wort from the lauter-tun is around 73 °C. The wort reaches boiling point at 93 °C in the smaller kettle and 99 °C in the larger kettle. The maximum wall temperature on the wort side is 107 °C. As subsequent processes involve cooling, it is necessary to lower the temperature using a reduction system. By preheating the wort before it enters the wort kettle, the amount of heat required for boiling can be reduced, as the temperature is raised from approximately 73 °C to 85–90 °C or higher. This presents an opportunity for heat recovery.

The findings of the study indicate that fuel consumption at Meta Abo Brewery is significantly high compared to similar breweries. This highlights the immediate need for fuel-saving measures. Energy-saving measures related to boilers and steam distribution have been summarized and provided in Table 7.

**Table 7:** Fuel saving measures, annual cost of saving, Implementation cost & Simple payback period for Meta Abo brewery.

S/N	Energy saving measures	Annual Thermal Energy Saving (MJ)	Annual Fuel Saving in (Liter)	Annual cost of saving (Birr)	Implementation cost (Birr)	Simple Payback Period (Year)	Recommendation
1	Adjusting Air-fuel ratio for the two boilers	2,193,080	56,892	1,028,038.00	300,000.00	0.3	Immediate
2	Fitting Economizer for boiler no 1	1,129,460	29,300	529,451.00	550,000.00	1.04	Immediate
3	Re-insulating of steam pipes having poor insulation	1,362,638	35,349	638,768.54	75,000.00	0.12	Immediate
4	Removing Steam leakage from steam pipes	1,218,660	31,614	571,265.00	100,000.00	0.18	Immediate
5	Installing new wort pre-heaters for the wort kettles.	11,092,996	287,770	5,200,000.00	4,500,000.00	0.87	Immediate
6	Buying and installing biogas line from WWTP to boiler room	5,759,862	149,420	2,700,000.00	1,000,000.00	0.37	Immediate
**TOTAL**		22,756,696	590,345	10,667,522.54	6,525,000.00		

#### 3.2.4 Energy saving measures prioritization and justification

The brewing industry has identified five priority measures for energy conservation. The first is fitting an economizer for boiler No. 1, which can save 1,129,460 MJ in thermal energy and 29,300 L in fuel. The second is installing new wort pre-heaters for wort kettles, which can save 11,092,996 MJ and 287,770 L in thermal energy and fuel. The third is adjusting the air-fuel ratio for two boilers, which can save 2,193,080 MJ and 56,892 L in fuel. The fourth is addressing steam leakage from steam pipes, which can save 1,218,660 MJ and 31,614 L in thermal energy and fuel. The fifth is re-insulating steam pipes with poor insulation, which can save 1,362,638 MJ and 35,349 L in thermal energy and fuel. These measures are crucial for energy conservation and cost savings.

#### 3.2.5 Impact of losses on boiler efficiency and specific actions to address them

Heat loss resulted from dry flue gas, H_2_ in the fuel, moisture in the fuel, air, surface radiation, surface convection, and boiler blowdown can significantly impact boiler efficiency. Dry flue gas loss occurs when the flue gas is not fully utilized to transfer heat before being discharged, resulting in reduced efficiency. To minimize this loss, enhance insulation and implement heat recovery systems like economizers or condensing heat exchangers. H_2_ in the fuel loss occurs when H_2_ in the fuel reacts with O_2_ from the combustion air to form water vapor, causing a loss of useful heat. A proper ratio of fuel to air and combustion control systems can ensure complete combustion and minimize excess water vapor formation. Melt loss in the fuel occurs when the moisture content absorbs heat during combustion without contributing to useful energy generation. Pre-drying the fuel before combustion and using fuel selection with lower moisture content or alternative fuel sources can help mitigate this loss. Air preheating and dehumidification systems can also help minimize moisture content in the combustion air. Surface radiation loss occurs when heat is emitted from the boiler's surfaces in the form of electromagnetic radiation. Enhanced insulation and refractory materials can help reduce radiation loss. Surface convection loss occurs when heat is carried away from the boiler's surfaces by circulating gases or liquids. Improving heat transfer surfaces within the boiler and applying heat-resistant coatings can help reduce convective heat losses. Boiler blowdown loss occurs during the blowdown process, leading to significant waste of heat energy. Implementing effective blowdown control strategies and heat recovery systems can help reduce these losses and improve boiler efficiency.

#### 3.2.6 Technical explanation and potential savings for the significant losses

The technical explanation of heat loss in a boiler can be divided into four categories: heat loss due to dry flue gas, heat loss due to H_2_ in the fuel, heat loss due to surface radiation, and heat loss caused by boiler blowdown. These losses can be reduced by improving insulation, adjusting the fuel-air ratio, using advanced combustion control systems, improving insulation on the boiler's surfaces, using refractory materials, and optimizing blowdown control strategies. The potential energy savings can range from 2% to 5% depending on the insulation quality and operating conditions. Heat recovery systems can capture and utilize heat from the blowdown water, reducing heat loss and allowing it to be used for preheating feed water or other processes. Overall, these measures can lead to significant energy savings, ranging from 5% to 10%, depending on the specific boiler type and operating conditions.

### 3.3 Carbon footprint estimation

The brewery contributes to greenhouse gas (GHG) emissions through various activities involving combustion and non-combustion processes. To determine the GHG emissions resulting from fuel consumption, the fuel quantity, gross calorific value, and emission factor were multiplied. Unfortunately, country-specific emission factors for the fuel oils used in electricity and thermal energy generation were not available. Therefore, emission factors from the Intergovernmental Panel on Climate Change (IPCC) 2006 guideline were utilized for estimating the GHG emissions from energy generation fuels. By applying Equation (17), the GHG emissions for the year 2015 were calculated, resulting in approximately 10,552 tons of CO2 equivalent. The emissions for other years were computed and presented in Table 8 and Table 9.

**Table 8:** Fuel consumption for boiler and associated GHG emissions in Meta Abo Brewery.

Year	Consumption Boiler Fuel, L	Net Calorific Value, TJ/ton	Emission Factor, ton CO_2_/TJ	GHG Emission, ton CO2 Eq
2014	3,075,266.00	0.0433	77	9156.00
2015	3,544,140.00	0.0433	77	10,552.00
2016	3,126,809.00	0.0433	77	9310.00
2017	3,656,454.00	0.0433	77	10,887.00
2018	3,837,092.00	0.0433	77	11,424.00

**Table 9:** Fuel consumption for electricity generators and associated GHG emissions.

Year	Consumption Boiler Fuel, L	Net Calorific Value, TJ/ton	Emission Factor, ton CO_2_/TJ	GHG Emission, ton CO_2_ Eq
2014	–	0.0417	74	0
2015	–	0.0417	74	0
2016	3,026,859.00	0.0417	74	8528.00
2017	3,789,230.00	0.0417	74	10,676.00
2018	4,001,235.00	0.0417	74	11,273.00

**Fig. 5:**
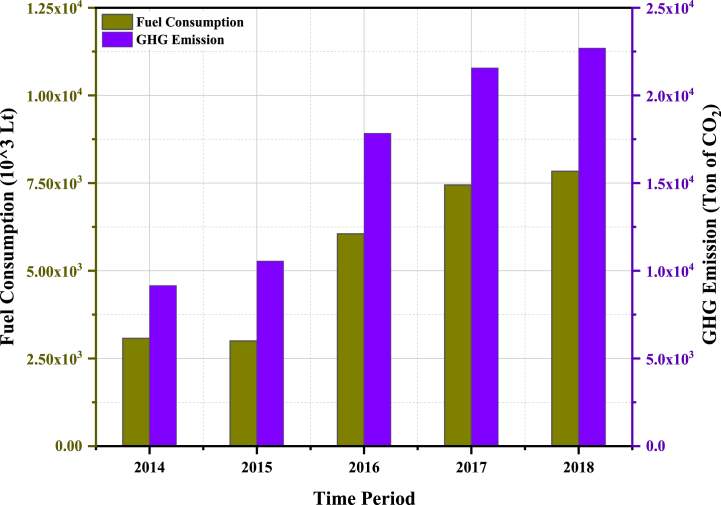
GHG emission trends of Meta Abo Brewery (2014–2018).

The findings of this study reveal that the GHG emissions observed at Meta Abo Brewery are comparatively higher than those of other renowned brewers, as depicted in Fig. 5. Previous studies indicate that a brewery with a capacity of 46 million hectolitres/annual emits only 446,000 tons of CO_2_ per year [48,49]. Fig. 5 demonstrates that in 2018, the GHG emissions from Meta Abo Brewery amounted to 22,697 tons of CO_2_. These higher emissions can be attributed to the energy sources utilized and inefficiencies within the brewery. The carbon footprint in the brewery industry varies based on various factors, including brewery size, packaging materials, and energy consumption. For instance, Alexander L. Bowler and colleagues reported a carbon footprint of 165 g of CO_2_ equivalent per liter of beer for the brewing process [50]. Furthermore, research conducted by The Climate Conservancy in 2008 [51] and Talve in 2001 [52] indicated carbon footprints of 58 and 240 g of CO_2_ equivalent per liter of beer for the brewing process, respectively. Similarly, Garnett in 2007 [53]and Koroneos et al. [54] reported carbon footprint values of 100 and 310 g of CO_2_ equivalent per liter of beer for the brewing process. The carbon footprint values reported in these studies indicate that the breweries examined showcased medium carbon emissions.

The brewery industry can effectively reduce carbon emissions through the implementation of technology and management strategies. Innovative approaches, such as transition engineering and participatory back casting, enable companies to define strategic pathways for enhancing energy efficiency [55]. Conducting cleaner production audits and implementing technological advancements can result in reduced energy consumption and emissions [56]. One environmentally friendly method is anaerobic digestion, which converts waste materials generated during beer production into biogas, leading to a significant decrease in carbon dioxide emissions. It is crucial to monitor indoor air quality in craft breweries to mitigate the impact of fermentation CO_2_ release and unintentional venting during CO_2_ storage tank filling, as these factors can cause a significant increase in indoor CO_2_ levels [57]. Information systems also play a vital role in monitoring and optimizing production efficiency and resource consumption, making them valuable tools for promoting sustainability in manufacturing industries [58].

A full life cycle assessment conducted by the Beverage industry also showed that a brewery with a capacity of 1.62 million hectoliters emits approximately 10,000 metric tons of CO_2_ equivalent annually [40,59,60]. Hence, urgent steps must be taken to transition entirely to renewable energy sources for energy generation. The adoption of energy-saving initiatives identified in this study will contribute to the reduction of GHG emissions. Table 10 presents the potential annual reductions in thermal energy, fuel consumption, and CO_2_ emissions achievable through the successful implementation of the proposed measures.

**Table 10:** GHG reduction due to the implementation of energy-saving measures.

No.	Energy saving measures	Annual Thermal Energy saving(MJ)	Annual fuel saving (Liter)	tCO_2_-emission reduction
1.	Adjusting the Air-fuel ratio for the two boilers.	2,193,080	56,892	169.0
2.	Fitting Economizer for Boiler I	1,129,460	29,300	87.0
3.	Re-insulating of steam pipes having poor insulation.	1,362,638	35,349	105.0
4.	Removing Steam leakage from steam pipes.	1,218,660	31,614	94.0
5.	Installing new wort pre-heaters for the wort kettles.	11,092,996	287,000	
6.	Total	16,996,834	440,155	455

## 4 Conclusion

Meta Abo Brewery has undergone an energy audit, revealing significant energy consumption trends and potential for energy-saving measures. The audit identified areas of energy losses, including boiler efficiency, steam distribution lines, and fuel losses. By implementing specific measures such as adjusting the air-fuel ratio, fitting economizers, re-insulating steam pipes, and addressing steam leakage, Meta Abo Brewery can improve its energy efficiency and reduce its carbon footprint. This audit is crucial for sustainable development in the brewery sector, setting an example for other industries to adopt sustainable practices. Incorporating sustainable energy options like hydropower, solar energy, or wind power can decrease reliance on fossil fuels and make a positive impact on climate change. The assessment provides Meta Abo Brewery with a clear direction, highlighting the significance of prioritizing energy efficiency and renewable energy as fundamental elements for a sustainable future.

## 5 Recommendations

Based on the findings of this study, the following suggestions were put forward:

➢ The boilers currently operate with relatively lower efficiencies, measuring 79.2% and 80.9% respectively. To enhance boiler efficiencies, it is advisable to adopt the proposed energy-saving measures.
➢ The brewery currently relies on a diesel generator for electricity production, consuming approximately 6000 L of diesel per day. To reduce electricity costs, it is recommended that the brewery transition to a renewable energy source.
➢ Immediate implementation of energy-saving measures for boilers, steam lines, and motors is recommended. However, conducting a more in-depth analysis of the economic feasibility of the proposed energy-saving measures is necessary.
➢ Although this study estimated significant greenhouse gas emissions, a comprehensive life cycle assessment is required to evaluate the holistic greenhouse gas emission impact of the brewery.

## Funding

No funding was obtained for this work.

## Ethical approval and consent to participate

Not applicable.

## Data availability statement

The authors confirm that the data supporting the finding of this study are available at: https://nadre.ethernet.edu.et/record/3882/files/ENERGY%20AUDIT%20AND%20ASSOCIATED%20CARBON%20FOOTPRINT.pdf?download=1.

## CRediT authorship contribution statement

**Eba Adino:** Writing – original draft, Visualization, Validation, Software, Methodology, Investigation, Formal analysis, Data curation, Conceptualization. **Mikiyas Abewaa:** Writing – review & editing, Writing – original draft, Methodology, Formal analysis, Data curation, Conceptualization. **Amare Tiruneh:** Supervision, Data curation, Conceptualization.

## Declaration of competing interest

The authors declare that they have no known competing financial interests or personal relationships that could have appeared to influence the work reported in this paper.
